# Clinical Sexologists’ Perceptions of the Potentials, Downfalls, and Best Practices for Digitally Delivered Therapy: A Lesson from Lockdown Due to COVID-19 in Portugal

**DOI:** 10.3390/bs13050376

**Published:** 2023-05-04

**Authors:** Ivanilda B. Costa, Andreia A. Manão, Patrícia M. Pascoal

**Affiliations:** 1 HEI-Lab, Digital Human-Environment Interaction Labs, Lusófona University, 376, Campo Grande, 1749-024 Lisboa, Portugal; 2 Sociedade Portuguesa de Sexologia Clínica, Rua 1º de Maio Nº 2, 5300-236 Bragança, Portugal

**Keywords:** e-health, clinical sexology, sexual medicine, Internet intervention, content analysis, COVID-19

## Abstract

Professionals who work in clinical sexology intervene in situations related to mental health, such as sexual dysfunctions and marital conflicts, often with vulnerable populations, e.g., people with chronic illnesses or trans people. In this work, we wanted to understand the perceptions these professionals have about using Internet interventions and how they perceive—based on their COVID-related experience and the reflections it brought about non-face-to-face interventions—the use of online interventions. During the first lockdown due to COVID-19 in Portugal, we used an online survey and collected answers from 39 Portuguese sexual health professionals to open questions about the use of Internet interventions. The data were analyzed following the summative content analysis procedures. Our results showed that sexual health professionals had several difficulties in clinical practice during the lockdown period, such as the perception that sexuality moved to the backseat in people’s lives. Even so, they stated that Internet interventions have several advantages, such as easy accessibility and excellent promotion of social justice. However, disadvantages were also pointed out. The current study allowed us to understand clinicians’ perception of the impact of the pandemic on sexual healthcare access and brought recommendations for good practice of sexual medicine with e-health.

## 1. Introduction

Despite the possibilities of e-health and telemedicine, most clinicians still present negative attitudes toward using technologies to deliver patient care [1]. The COVID-19 pandemic helped to change this widespread attitude because this pandemic, one of the biggest health crises of the 21st century, had implications for the economy and the healthcare system [2]. The COVID-19 outbreak forced a complete reorganization of healthcare systems, which resulted in, among other things, remote care, and monitoring of disease [3]. It also meant that other clinical conditions were attended to with the implementation and use of digital technologies and the possibility of developing assistance through telemedicine. This possibility was made possible for different clinical conditions, some of them in the field of emotional and psychological problems, because of the accentuated negative impact that COVID-19 has had on psychological well-being [4,5].

Concerning mental health, several studies have demonstrated that the COVID-19 pandemic has negatively impacted several areas of people’s lives [6] and people with pre-existing disorders, are more vulnerable to experiencing a worsening of their mental health [7,8]. In this way, various psychological problems emerged, including irritability [7], frustration [8], fear of/and uncertainty during the COVID-19 pandemic [7,8], anxiety and depression [9,10], eating disorders [11], sleep disorders [12], and symptoms of post-traumatic stress disorder [6]. Furthermore, the same negative implications seemed to also happen in sexual health as, for example, in a longitudinal study [13], the results showed that levels of sexual distress were higher during the COVID-19 lockdown period when compared with the post-confinement period.

Sexual health is characterized by “a state of physical, emotional, mental, and social well-being about sexuality; it is not merely the absence of disease, dysfunction or infirmity […] requires a positive and respectful approach to sexuality and sexual relationships, as well as the possibility of having pleasurable and safe sexual experiences […]” [14]. Regarding sexual health, the impacts of the COVID-19 lockdown period can vary since several variables can serve as moderators, such as if the person lives with a partner, lives alone, or has children [15]. In this sense, studies suggest that virus containment measures (e.g., lockdown, social distancing, and isolation) may have added additional challenges for people with preexisting sexual dysfunction to cope with their condition (e.g., more exposure to sexual desire discrepancy), as well as possibly leading people to experience sexual dysfunction for the first time [2].

Some authors [16] have provided some light on what could be a potential challenge to the practice of sexual medicine during the time of COVID-19, mainly the alterations in professional demands (e.g., the allocation of some sexual health professionals to help in the COVID-19 intervention), social adaptations (e.g., families adaption to teleworking and helping their children’s school needs), adaptations in the dimension of the relationship (couples dynamics may change), and adaptations in individual dimensions (e.g., how people emotionally deal with the lockdowns, increases in sexual difficulties, and poorer sexual functioning). This view has been supported by research in different geographies. For instance, a systematic review of the literature about the effects of lockdown on sexual function has demonstrated that sexual function worsened and sexual frequency diminished, especially for women [17]. 

Reduced sexual function is not the only effect of COVID-19-related lockdowns. Studies show that sexual and/or gender minorities were particularly affected [18]. This is because minority stressors (e.g., discrimination and violence; [19]) were exacerbated during the COVID-19 pandemic [20]. Thus, sexual and/or gender minorities, such as those living in unstable housing, may not have access to the specialized services they used to have [21]. In this way, people, often clients of sexology clinical services (e.g., trans people), were left vulnerable. 

Thus, adopting strategies that provide people with the necessary support they need when prevented from accessing face-to-face health contexts has become essential. For example, the European Society for Sexual Medicine claimed that “online sexual health interventions can have beneficial effects on sexual and relationship outcomes” [22], reinforcing that online-delivered clinical services may help to overcome the shortage of available resources in sexual health.

However, to better understand if these recommendations and practices have a long-term impact, the question arises: “What are the professionals’ perceptions of using digital technology in their practice?” Considering the technology acceptance model (TAM; [23]), some factors can influence the adoption/acceptance of technologies in various settings. It has been explained that the use of new technologies is influenced by perceived usefulness, which is defined as the extent to which a person believes that using technology will improve his/her performance at work and the perceived ease of use, which is characterized by the ease that the person thinks the use of technology will have. Six processes explain the relationship between perceived usefulness and the use of technologies: (1) computer self-efficacy (i.e., the degree that the person believes that they can perform a task on a computer); (2) perception of external control (i.e., the degree that the person believes that the technological resource supports the task); (3) computer anxiety (i.e., the degree that the person fears when faced with the possibility of carrying out a task on a computer); (4) computer playfulness (i.e., the degree of spontaneity in computer interactions); (5) perceived enjoyment (i.e., the degree of perceived enjoyment when using the computer to complete a task); and (6) objective usability (i.e., the effort required to complete a task when compared with other ways to complete it). Thus, TAM explains healthcare professionals’ reactions to using new technologies in clinical interventions [24]. In the context of mental health, this model is in part supported by preliminary work about Portuguese psychologists’ knowledge, training, use, and attitudes regarding Internet interventions [25]. The results of this study showed that the psychologists were not familiar with or did not have prior experience with using digital technology in their clinical practice. However, they also found that despite the psychologists’ attitudes toward Internet interventions ranging from slightly negative to neutral, they could find advantages in using these interventions (e.g., cost-effectiveness and accessibility). In this line, in the sexual health field, there is preliminary empirical evidence that sexual health professionals experienced the usage of digital technology in their practice during the pandemic as a vital resource to support and develop clinical interventions [16]. However, to the best of our knowledge, there are no studies about specific perceptions, downfalls, and best practices for e-health therapy according to sexual health professionals. Therefore, this exploratory paper aims to fill these gaps in the literature.

Because this is an exploratory qualitative study developed in a new world context, we used an inductive approach [26,27] that is not limited to the rules imposed by structured methodologies. This way, we do not have a prior hypothesis and we followed established guidelines for exploratory qualitative research that recommend presenting an open-ended research question [28].

Thus, the current study aims to answer the main research question: “What are Portuguese sexologists’ perceptions about the use of Internet-related technologies to deliver clinical sexology healthcare?” further exploring clinical sexologists/specialists in sexual medicine perceptions of using new technology in clinical practice. Namely, based on their experience during the first lockdown period, we explore the perceived difficulties, advantages, and disadvantages of online interventions and their suggestions to ensure the best practices on Internet interventions aimed at sexual health. 

## 2. Methods

### 2.1. Research Design

The research aimed to explore clinical sexologists’ perceptions of using digital technology in their practice based on their experience during the 1st lockdown related to COVID-19.

For this purpose, we developed an anonymous cross-sectional study that collected online data from a convenience sample of certified clinical sexologists/specialists in sexual medicine through professional newsletters from associations such as the Portuguese Association of Psychologists, and Portuguese Association of Family Planning.

To develop the study, we followed a participatory design [29] involving the board of directors of the Sociedade Portuguesa de Sexologia Clínica (SPSC—Portuguese Society of Clinical Sexology) in the survey design. Based on their experience, the specialists agreed that an online survey would be an adequate methodology to collect anonymous data and that this approach would elicit more participation than face-to-face methodology. However, they also advised to refrain from collecting too much socio-demographic information that, despite the anonymous nature of the study, it could be easy to identify any participant. Therefore, the members of SPSC reviewed the survey protocol for adequacy, relevance, and length. After there was a consensus, a final version was made available online. The study had four parts: informed consent, sociodemographic data, questions on COVID-19 and sexual health, and the use of the Internet means for clinical work in sexual health. To answer the main research question, “What are Portuguese sexologists’ perceptions about the use of Internet-related technologies to deliver clinical sexology healthcare?”, four open questions were formulated: (1) what personal difficulties have you encountered in the practice of clinical sexology after the start of the pandemic (whether online and/or face-to-face)? (2) Regarding Internet-based intervention in clinical sexology, what is its advantage? (3) Regarding Internet-based intervention in clinical sexology, what are its disadvantages? (4) Regarding Internet-based interventions in clinical sexology, what are the necessary measures and conditions to ensure good practice? Because the study was advertised through professional newsletters and all eligible participants were invited, we cannot determine how many people were reached and how many read the newsletters through which the study was advertised. Therefore, we cannot determine the compliance rate.

### 2.2. Participants

A non-probabilistic convenience sample of 39 Portuguese sexual health professionals were included in the study. A total of 39 participants agreed to participate. The participants’ age ranged from 32 to 73 years of age, with a mean of 48.59 (SD = 10.11) years of age, 24 (66.7%) identified as women, and 15 (23.3%) identified as men. In addition, there were 15 physicians (38.5%; 7 psychiatrists, 3 urologists, 3 gynecologists, and 2 did not specify), 22 clinical psychologists (56.4%), and 2 nurses (5.1%). A total of 5 participants (12.8%) usually worked only in public settings, 14 (35.9%) worked exclusively in private settings, and 20 (51.3%) worked in both contexts (public as well as private settings).

### 2.3. Ethical Consideration

The authors followed all principles and guidelines in the Helsinki Declaration and those enumerated in the European Textbook on Ethics and Research [30,31]. Informed consent was obtained from all participants. Furthermore, the deontological procedures were positively reviewed by the Portuguese Association of Psychologists. 

### 2.4. Data Collection Methods

In Portugal, the COVID-19 pandemic officially began on 2 March 2020. Confinement was legally imposed on the general population on 18 March 2020. In total, the state of calamity in Portugal lasted 173 consecutive days, with different virus containment measures [32].

Thus, since mandatory confinement was already imposed, online data was collected from 22 March until 4 May 2020. The link to the web page where the study was implemented was advertised through the newsletters of recognized associations of professionals in the field of clinical sexology or sexual medicine in Portugal (e.g., Sociedade Portuguesa de Sexologia Clínica; Sociedade Portuguesa de Andrologia; and Associação para o Planeamento da Família) with an invitation to participate. After reading the informed consent form, where information disclosing the research team, the purposes of the research, the contact of the PI, and the measures for data protection and storage, such as the guarantee that only the research team would access the data collected, the participants could agree to participate in a study about their perceptions of sexual health during the COVID-19 pandemic. The inclusion criteria were: (a) comprehension of the Portuguese language, (b) having an education (certified degree) and supervised training in clinical sexology (including sex therapy and sexual medicine), and (c) being an active practitioner of clinical sexology since before the pandemic. On the first page, minimal socio-demographic data were collected. Afterwards, there was a page with open questions about how COVID-19 had affected people’s sexual health, which was translated into a previously published manuscript [16]. The last part had questions about clinical sexologists’ perceptions of using Internet interventions, defined as therapeutic interventions operated via secure platforms, mobile apps, or video-conference services that aim to provide health and mental health-related assistance. No IP or geolocation information was saved. The database was archived in a digital database protected through a password and is only accessible to the research team.

### 2.5. Data Analysis

The analysis followed the summative content analysis of Zhang and Wildemuth (2009) [33], a widely used qualitative research technique within health and social scientific investigations [34].

Coding was performed in parallel by two researchers (I.B.C. and A.A.M.). Our analysis followed the following procedure: (1) we started by completing the initial familiarization with the data through repeated transcript reading to gain a general idea of the content; (2) we defined the unit of analysis for the transcriptions; (3) we then examined the transcriptions to define the coding schema; (4) we tested our coding scheme on a sample of text of our transcriptions to test the consistency and clarity of our category’s definitions, with the level of coding consistency as high; (5) we coded all the transcriptions; and (6) we rechecked our coding consistency to ensure that the consistency was maintained. At an early stage of the analyses, the codes produced were semantic, and in the posterior phase, the medium responses became more latent (more in-depth). After reaching a consensus, a senior author (P.M.P.) checked if the data fit the final proposal of categories and discussed this with the main coders until a consensus was reached. Finally, we summarized and narratively reported the results presenting frequencies for categories and each code.

## 3. Results

Participants’ responses ranged from concise responses (e.g., a single word, for example, “accessibility”) to medium responses (e.g., two or three sentences. Examples can be found below, ad verbatim). 

In terms of the participants’ response length, it varied in the four questions. The question about the difficulties encountered in the practice of sexology after the start of the pandemic, globally, had lengthier answers, as participants provided more examples to illustrate their point of view. They were, therefore, richer and revealed more engagement with this topic. The question regarding the advantages of Internet-based intervention in clinical sexology had the shortest answers, revealing less engagement with this topic. The questions about the disadvantages, necessary measures, and conditions to ensure good practice demonstrated similar lengths and details.

Most of the participants reported having only one or two difficulties using Internet interventions, and among the people who reported three or more difficulties, we found mainly gynecologists and clinical psychologists. 

Most participants also reported at least three advantages of using interventions online. Even so, these participants also pointed out at least three disadvantages.

Regarding the conditions for good practices in online interventions, approximately 40% of the respondents presented three or more conditions necessary for the good practice of online interventions. More than 85% of them work as psychologists, with two psychiatrists and two nurses giving three or more pieces of advice. 

When inspecting the respondents’ answers and considering their professional experience, we found no pattern in the answers that linked the years of experience and adherence to online clinical interventions. 

The results’ presentation follows the order in which the questions were presented. 

### 3.1. “What Personal Difficulties Have You Encountered in the Practice of Clinical Sexology after the Start of the Pandemic (Specify Whether Online and/or Face-to-Face)?”

From the identification of the codes related to difficulties with online intervention, we defined five categories, namely, “Sexuality is not important?” (n = 8), “Privacy” (n = 14), “Technology” (n = 9), “Interferences/shortcomings in the therapeutic process” (n =11) and “Work/Family balance” (n = 1). 

In the category “Sexuality is not important?” professionals reveal that during the COVID-19 pandemic, sexuality took a back seat in people’s lives. In this way, three codes were identified: (1) “Cancellations” (e.g., “People cancel sessions” and “The significant reduction in follow-up, because they consider that they prefer face-to-face”: n = 2); (2) “Postponing” (e.g., “Clients prefer in-office intervention; some postponed interventions until face-to-face intervention were possible”: n = 1); (3) “Fewer cases” (e.g., “total reduction of new cases; for now, people are more focused on the virus”: n = 2). Moreover, some professionals stated that they stopped practicing clinically (e.g., “The clinic closed”: n = 1), which also promoted the category “Activity suspended” (n = 2). 

Regarding the category “Privacy,” we defined two codes: (1) “Client” (n = 5) and (2) “Therapist” (n = 4). The first is related to concerns regarding client’s privacy (e.g., “Clients’ privacy: Do not feel comfortable having a clinical session at home with the partner nearby” and “Appointments interrupted by family members/other people”), and the second is related to concerns with professionals’ privacy (e.g., “Privacy to develop clinical sessions” and “I don’t have enough [sound] safety conditions in the hospital consulting rooms”).

Regarding the category “Technology”, professionals seemed to have difficulties at two levels, which led to the development of the following codes: (1) “Connection” (n = 5) and (2) “Safety” (n = 4). The first refers to problems related to the Internet connection (e.g., “Unstable Internet access”), and the second is related to concerns related to the security of new technologies (e.g., “Guaranteeing 100% confidentiality, as programs are susceptible to suffering piracy”).

Another category that was defined was “Interferences/shortcomings in the therapeutic process” (n = 11), which was composed of six codes: (1) “More challenging to establish a therapeutic alliance” (e.g., “Losses in the richness of the face-to-face therapeutic relationship”: n = 4); (2) “Less availability from professionals” (e.g., “I have to refer [other professionals] and many colleagues of psychology and therapy are not so available”: n = 3); (3) “Less non-verbal clues” (e.g., “I like the visual contact and need to properly assess facial expressions. And I also like my expression to be noticed. Even face-to-face, with the use of a mask this is difficult”: n = 4); (4) “People are less at ease” (e.g., “There is greater difficulty in expression [their feelings and thoughts], due to physical distancing”: n = 1); (5) “The reasons to seek help are mode diffuse or more holistic” (e.g., “Women became more vague in their complaints”: n = 1); and (6) “Difficulties to conceal couple’s agendas” (e.g., “When needed to work with a couple and only one is available”: n = 1).

Last, the professionals mentioned that another difficulty they faced in online consultations during the COVID-19 pandemic was the appointment schedule due to the overlapping of working hours with family time (n = 1). In this way, the category “Work/ family balance” was defined with one code, namely, “work schedule” (e.g., “Manage a stable schedule—family at home”). Another difficulty that the professionals felt was “Excessive work after hours”, which impacted family time. 

### 3.2. “Regarding Internet-Based Intervention in Clinical Sexology, What Do You Consider an Advantage of Using It?”

From the identification of the codes, one of the categories is defined as “Improvement access” (n = 22). Defining this category aimed to detail the perceptions of mental health professionals who showed that online sessions, for various reasons, allowed greater access to people with different conditions/characteristics. As a result, four codes were defined: (1) “Less financial burden” (e.g., “Accessible”: n = 8); (2) “Less administrative burden” (e.g., “More practical”: n = 2); (3) “Time-effective” (e.g., “Flexibility [...] avoiding traveling, the person can have an appointment without having to go to a clinic”: n = 3); (4) “Better scheduling” (e.g., “Allows flexibility of schedule on the part of those who access”: n = 4); and (5) “More social justice” (e.g., “It allows us to reach a larger audience of patients, who would otherwise not be able to seek help”: n = 5). Thus, these professionals reveal that the advantages of online interventions are lower prices for clients, less bureaucratic processes to be followed, more efficiency in the time consumed in the client’s day, and equal access to all people who use the service, regardless of geographic distance and the physical condition of the person, among others. 

The other category defined was “Improvement in therapeutic practice” (n = 29), where sexual health professionals addressed various topics through which they believe online interventions could benefit the client’s therapeutic process. Four codes were defined: (1) “More geodiversity” (e.g., “Allows people in geographical areas without an offer to access to clinical service”: n = 8); (2) “More self-disclosure” (e.g., “Some in-person restraint exposure. It removes some tension/anxiety regarding this point because, in the comfort of the home, the person can […] expose themselves to the therapist; the person feels less exposed” and “Fewer psychological and emotional barriers to overcome”: n = 14); (3) “More appealing for marginalized populations” (e.g., “It also allows for greater disclosure on topics such as gender and sexual orientation” and “Easier communication […] reduction of stigma”: n = 3); and (4) “Flexibility” (e.g., “Access to services for people who do not have clinical sessions near them”: n = 4). 

### 3.3. “Regarding Internet-Based Intervention in Clinical Sexology, What Are Its Disadvantages?”

Considering online interventions’ disadvantages, one category was “Limitations in the therapeutic process” (n = 69). According to the professionals’ perceptions, eight codes were defined: (1) “Creating a therapeutic alliance” (e.g., “Loss of the potential of the face-to-face therapeutic relationship; process artificiality”: n = 14); (2) “Maintaining a therapeutic alliance” (e.g., “Distancing in the therapeutic relationship that may have implications for the outcome of therapy”: n = 9); (3) “More difficult to work with couples” (e.g., “The main thing is for the couple to be alone at home, and to be able to organize the day to have time available”: n = 6); (4) “Discomfort with the setting (e.g., “Virtual context of carrying out the intervention, with a certain constraint”: n = 3); (5) “Impossible to make the physical observation” (e.g., “Need for complementary observational exams, cannot be neglect”: n = 5); (6) “Less access to non-verbal clues” (e.g., “Difficulty in assessing customers in their non-verbal and postural language”: n = 7); (7) “Less access to non-verbal clues” (e.g., “No complete observation of body posture”: n = 7); (8) “More challenging to work with multidisciplinary” (e.g., “Multidisciplinary assessment and networking are more difficult; at least for now, medical colleagues are less available”: n = 2); and (9) “Adherence/commitment” (e.g., “Less preference for the virtual resource given the specificities of the sessions”: n = 15). Thus, the professionals mentioned that the significant disadvantage of using online interventions was related to limitations in establishing/maintaining the therapeutic alliance and the difficulty in perceiving non-verbal clues from a digital screen. Furthermore, the impossibility of making physical observations was also mentioned, an essential test in sexual health. In addition, they revealed that it is more challenging to work with couples since it is difficult, for example, for the couple to be alone at home without outside members during the clinical session. In addition, it was mentioned that patients are often uncomfortable with having the sessions at home.

Another category that professionals addressed as a disadvantage was related to “Technological issues” (n = 14), which contains two codes: (1) “Equipment/connection problems” (e.g., “Network and connection failures, which sometimes compromise some precise and momentary intervention”: n = 11) and “Media literacy” (e.g., “Not everyone is aware of or comfortable with new technologies” and “People without resources for new technologies also do not access”: n = 3).

The other category defined was “Ethical issues” (n = 13), where professionals address that some disadvantages are related to ethical issues, as is the case with the following two codes: (1) “Privacy” (e.g., “There is a greater possibility of blurring [being interrupted]”: n = 7); and (2) “Confidentiality of data” (e.g., “Ethical issues/data retention”: n = 3).

Finally, professionals also mentioned that a disadvantage of using online interventions is the “Negative impact” (n = 3) that they can have on clients, such as (1) “Less clarity about what a clinical setting is” (e.g., “Clinical setting bias”: n = 2) and (2) “Isolation reinforcement” (e.g., “Reinforcement of isolation”: n = 1).

### 3.4. “Regarding Internet-Based Interventions in Clinical Sexology, What Are the Necessary Measures and Conditions to Ensure Good Practice?”

Considering the measures and conditions professionals consider necessary to ensure good practice, we grouped their responses into five categories: (1) “Therapist skills” (n = 41); (2) “Technological capacity” (n = 5); (3) “Blended format” (n = 4), (4) “Ethical clarifications and procedures” (n = 17); and (5) “Based on empirical data that sustains/grounds its use” (n = 1).

Hence, sexual health professionals pointed out that, to have good practices in online interventions, the professional must have (1) “Personal characteristics” (e.g., “Creativity”: n = 4); (2) “Professional skills” (e.g., “Active listening ability”; n = 16); and (3) “Media literacy (e.g., “The therapist, in my opinion, should be familiar with the technology required for these approaches”: n = 7). In addition, sexual health professionals also point out (4) “Communication skills (e.g., “Good communication skills”: n = 4); and, finally, (5) “Education on online interventions” (e.g., “Reading/informing yourself about online interventions and ethics; the Order of Psychologists has training available so that you are not caught in a session with difficulties”: n = 10).

Another category that was defined as “Technological capacity” has two codes: (1) “Client” (n = 2) and (2) “Therapist” (n = 3). In this sense, professionals highlighted the importance of both the therapist being familiar with new technologies (e.g., “Mastering the use of apps”), but also the client being familiar (e.g., “Trying to understand if the person also understands (of technologies”). 

Even pointing out the advantages of using new technologies, mental health professionals showed that a necessary condition for their use is the complementarity with face-to-face sessions. This way, another category was defined: “Blended format (e.g., “Possibility of face-to-face as a complement”: n = 4).

Regarding the category “Ethical clarifications and procedures”, the results show us three codes: (1) “Storage” (e.g., “Carefully choose the platform you use [data privacy]”: n = 1); (2) “Safe technology” (e.g., “Privacy conditions and effective and secure technology”: n = 6); and (3) “Follow the deontological code of conduct” (e.g., “Rigorous in complying with ethical and deontological principles”: n = 10).

Finally, another category is defined as “Based on empirical data that sustains/grounds its use”, which has one code: (1) “Research into the implementation of online interventions.” The following is an example of this code: “Much research for our population before putting these methods into practice” (n = 1).

## 4. Discussion

In this manuscript, we aimed to explore the perceptions of clinical sexologists about the use of Internet-delivered healthcare based on the experience that the COVID-19 pandemic has brought in this field [33] and used a qualitative approach by delivering a set of open questions that were advertised in professional newsletters in the field of sexual medicine and clinical sexology.

Overall, the participants’ answers showed that the COVID-19 pandemic was an opportunity to make room for experimenting for the first time with new technologies in the context of online interventions. This result is congruent with other investigations [16] that state that most Portuguese psychologists do not have training or experience in using Internet interventions. Therefore, the pandemic came as an opportunity for experimentation. Taken together, our results also demonstrate that there are specificities in the use of Internet-delivered interventions in sexual health that should be considered, such as the difficulties of implementing a multidisciplinary approach and the asset that Internet interventions are for vulnerable groups due to sexual identity or orientation. 

Considering the first question: “What personal difficulties have you encountered in the practice of clinical sexology after the start of the pandemic (specify whether online and/or face-to-face)?”, our results seem to be in line with research [35,36] that has highlighted that people that were in lockdown and with a partner significantly reduced their participation in sexual intercourse, and this may account for the lowering of patients and requests for consultation reported by our participants. If people take part in less sexual activity, they may be less frequently facing their sexual problems. Complementarily, it can also mean that for those who already had a sexual problem, the lack of privacy due to the lockdown that forced people to stay at home may have caused people to refrain from seeking help which could have contributed to the worsening of their problems and the rise in sexually-related conflicts. Furthermore, working in an online format seems to have promoted overwork after hours, reducing pleasure and family time and thus increasing social distancing, which may have influenced the reduced role of sexuality in the periods of confinement. On the other hand, in the same way that professionals may feel reluctant to use new technologies to support clinical interventions, it can also be that clients who already have contact with face-to-face therapy report having less desire to try therapy in an online format [37] especially if we take into account that professionals report that they perceived that people gave less importance to their sexual problems during the pandemic. 

The existing literature demonstrates that client-related alliance scores on Internet-delivered interventions are equivalent to alliance ratings on face-to-face therapy [38]. However, our participants report difficulties establishing a therapeutic relationship when it is mediated by technology. It would be essential to enquire if, from the client’s point of view, a relationship can be established regardless of the modality of the clinical session. There seem to be myths considering the impossibility of establishing a therapeutic alliance online [38], which compromises the exploration of e-health. The pandemic created the need to overcome these myths. However, it will be interesting to understand if this opportunity translates, or not, into more flexible attitudes towards technologically mediated health services. 

This reluctance to use telemedicine and online solutions in clinical sexology may be worsened by the technical difficulties that our participants report. As the TAM continuously points out, easiness to use is one factor that predicts the attitude and the intention to use technology. A recent study pointed out that the lack of privacy at home predicted a higher rate of interrupted treatments in clinical sessions, which can negatively impact the client’s therapeutic process [25]. Furthermore, technological problems were a source of concern in the clinical sessions. People with less access to technology may have weaker therapeutic experiences, constantly struggling with an unstable Internet connection. Thus, people who struggle with unstable Internet connection may perceive that Internet solutions may be less effective due to connection problems and connection instability.. In that case, they will likely use it less or prefer not to use it. The quality of one’s Internet connection depends on the financial agreement with the company that provides this service. It can also depend on the living location of the person. Therefore, although Internet solutions may help overcome access to treatment barriers, this access is still grounded in social and financial inequalities. It should be noted that technology is rapidly evolving and that people’s digital literacy must be considered. In other words, if technology continues to evolve and people do not acquire digital skills simultaneously, this can lead to digital exclusion.

The security of Internet applications was another concern regarding the need for more confidence in the privacy of web applications. The sexual health professionals reported that people feared that the information they gave would be hacked, or the professionals themselves reported that they feared being recorded. These fears translate not only a lack of confidence in technology but also a lack of confidence within the therapeutic context, an unexplored area in the e-health literature.

Finally, the professionals mentioned that another difficulty they faced was appointment schedules due to overlapping working hours with family time. This is consistent with other studies that demonstrate that the COVID-19 lockdown created an imbalance in care responsibilities for people who continued to work [39], especially for people who identified as women [40], which may indicate that the pandemic context compromised female therapists’ work. In addition, these obstacles related to Internet-delivered clinical services may have contributed to a worsening of sexual problems of people previously participating in face-to-face treatment because stopping sexology clinical sessions may lead to adverse outcomes in sexual health (e.g., sexual dysfunctions [41]).

In short, the experience of clinical sessions in an online format could have been more favorable for patients with social advantages, excluding people with difficulties in using the Internet, who are usually from older age groups, minority ethnicities, with lower levels of education, and people with worse housing conditions (no privacy). In other words, this can lead to the exclusion of already marginalized groups. This calls for an intersectional look at the use of e-health. 

Considering the second question: “Regarding Internet-based intervention in clinical sexology, what do you consider to be an advantage of using it?”, the sexual health professionals highlighted several positive experiences, such as increased efficiency in administrative tasks, no travel time, reaching more areas of the country, and more flexibility in scheduling appointments. In other words, since e-health allows people to overcome location and scheduling constraints and be more flexible, it can be considered a win–win situation—both for the therapist who manages to reach people from different areas and for clients who, despite their conditions/locations, can have access to the clinical session. That way, e-health may increase health service efficiency and user satisfaction. 

Furthermore, the professionals refer to “more social justice” as another code illustrating, for example, that people with disabilities can more easily attend clinical sessions since there are fewer physical barriers to e-health. Furthermore, regarding, for example, gender minority people, COVID-19 led to new stressors (e.g., living full time with a non-supportive family) [42]. Choosing to give sexology sessions in an online format helped to assist this population. In other words, online interventions can be a specificity of interventions in sexology, responding to structural problems such as those faced by minorities (e.g., social inequalities and disparities in clinical follow-up in health and sexual health) [43]. Future studies explicitly focused on intersectionality should be carried out since it is the only way to understand opportunities and inequalities in the health and sexual health sectors.

Considering the third question: “Regarding Internet-based interventions in clinical sexology, what do you consider to be the disadvantages of using it?”, the professionals mentioned that the significant disadvantage of using online interventions was related to limitations in establishing/maintaining the therapeutic alliance, as well as the difficulty in perceiving non-verbal clues from a digital screen. The impossibility of making physical observations was also mentioned, which is, for some professionals (e.g., gynecologists), an essential assessment tool in the field of sexual health. However, it is important to emphasize that this could be a limitation depending on the reason for the consultation. In some instances (e.g., if the person is looking for a sexologist to work on negative beliefs related to non-normative sexual orientation), the impossibility of making physical observations may not be a problem. It was the gynecologists who indicated more disadvantages in using e-health therapies. In addition, the experience of professionals demonstrates that it is more demanding to work with couples since the difficulties of concealing agendas or having privacy are boosted. It was also mentioned that online interventions could increase the experience of isolation, both for mental health professionals and their clients. Therefore, it becomes necessary to analyze and understand the best practices to overcome these difficulties, partially addressed in the fourth question.

Thus, considering the fourth question: “Regarding Internet-based interventions in clinical sexology, what do you consider to be the necessary measures and conditions to ensure good practice?”, some answers referred to factors that may also play a role for both online and face-to-face interventions (i.e., “Personal characteristics”; “Professional skills”; and “Communication skills”).

Sexual health professionals highlight that to provide a good e-health service, it is important for the professionals to be familiar with online interventions and they should receive training. Moreover, sexual health professionals strongly recommend that therapists be aware of the online platform they use to avoid being hacked and that they follow the guidelines and codes of ethics for working with their clients. In this line, it is recommended that mental and sexual health professionals undertake training on e-health therapies. This training is available at the Portuguese Psychologists Association and various associations of psychology. Health professionals must strongly consider this. The answers of our participants stress that there must be bidirectional movement between the professional who seeks training and the associations that represent them by providing updated knowledge and guidelines in this field. 

Our results confirm that adopting clinical sessions in an online format may depend on several variables, such as the perception that the technologies are helpful and that it is easy to work with them. In particular, the possibility of Internet failure during clinical sessions was one of the most significant disadvantages that the professionals mentioned. In this way, we can see that the perception of external control was a variable that significantly impacted the perception of sexual health professionals in using new technologies. Thus, our results are in line with the TAM model. Even so, sexual health professionals highlight that it is essential for professionals to be trained in new technologies, not neglecting the possibility that, if they do not know how to work with them, they have the training to do so. 

### 4.1. Limitations

The limitations of the current study need to be considered. Firstly, we need to acknowledge that there may be researcher bias in developing the open questions and interpreting the results as the primary research team favors using digital technology to deliver healthcare. In addition, the definition of Internet intervention presented is comprehensive and may include different forms of e-health (apps and video-conferencing). Therefore, one should be cautious when reading the results of this study as they may not portray the views of all clinical sexologists and people who act on sexual medicine. Furthermore, it is unclear if the people who answered the survey were only answering about the use of video conferencing or if they were also considering the use of other forms of Internet interventions, such as the use of platforms for self-guided or blended therapy as the participants mainly did not specify to which specific service they were referring. Moreover, the fact that we did not collect data or proxies to determine new media literacy also hinders a better understanding of the professionals’ attitudes, as these may be moderated by new media literacy. Finally, the necessary caution taken to ensure the anonymity of the data, with a small amount of socio-demographic data collected, prevented us from better understanding some patterns of the results, namely those concerning the geodiversity of the participants and their familiarity with e-health in their departments.

### 4.2. Future Research Directions

Recognizing the method allows full disclosure, but perspectives from different countries and healthcare systems may be helpful to better comprehend practitioners’ attitudes towards integrating Internet intervention in their practice. In addition, future studies should better specify which kind of Internet or digital service the participants are being questioned about, as attitudes may differ based on the e-health service delivered. Finally, we believe it is crucial to develop large-scale studies aimed at stakeholders and putative users to better understand their receptivity to this kind of intervention and based on TAM 3, what features would increase their intention to use these technologies.

## 5. Conclusions

In conclusion, the current study, developed in the context of the COVID-19 pandemic and its implications for healthcare access in Portugal [16,44], is a step towards developing knowledge about using new technologies in sexual health clinical sessions. Future research may help enhance the findings and determine variables such as barriers and facilitators of using new technologies in sexual health contexts. Our framework can guide researchers to develop guidelines, training, and more research to enlarge this promising area of online interventions.

This exploratory study on sexologists’ perceptions of the use of Internet interventions shows that professionals seem receptive to promoting the best use of digital health strategies but still recognize a lot of difficulties and disadvantages of this kind of intervention, with them tending to be favorable to a blended type of intervention that ensures patients and clinicians have real-time face-to-face contact. The need for specific education about the best ethical practices and strategies to overcome the disadvantages perceived by professionals, namely technical difficulties, is mandatory. Overall, professionals recognize the value of e-health to expand access to sexological healthcare to marginalized and stigmatized groups and individuals. Professional societies play a fundamental role in promoting the best ways to use e-health in clinical sexology and sexual medicine.

## Data Availability

We decided to collect only a few pieces of socio-demographic data; nevertheless, due to the small number of clinical sexologists/professionals in sexual medicine in Portugal, it could be possible to identify some people based on the limited data collected. Therefore, the authors have decided not to make the data available because the informed consent form stated that the data would only be accessed by the research team.

## References

[1] Rho M.J., Choi I.Y., Lee J. (2014). Predictive factors of telemedicine service acceptance and behavioral intention of physicians. Int. J. Med. Inf..

[2] Pennanen-Iire C., Prereira-Lourenço M., Padoa A., Ribeirinho A., Samico A., Gressler M., Jatoi N.A., Mehrad M., Girard A. (2020). Sexual Health Implications of COVID-19 Pandemic. Sex. Med. Rev..

[3] Mahmood S., Hasan K., Carras M.C., Labrique A. (2020). Global preparedness against COVID-19: We must leverage the power of digital health. JMIR Public Health Surveill..

[4] Vindegaard N., Benros M.E. (2020). COVID-19 pandemic and mental health consequences: Systematic review of the current evidence. Brain Behav. Immun..

[5] Monaghesh E., Hajizadeh A. (2020). The role of telehealth during COVID-19 outbreak: A systematic review based on current evidence. BMC Public Health.

[6] Bridgland V.M., Moeck E.K., Green D.M., Swain T.L., Nayda D.M., Matson L.A., Hutchison N.P., Takarangi M.K. (2021). Why the COVID-19 pandemic is a traumatic stressor. PLoS ONE.

[7] Gavin B., Lyne J., McNicholas F. (2020). Mental health and the COVID-19 pandemic. Ir. J. Psychol. Med..

[8] Vigo D., Patten S., Pajer K., Krausz M., Taylor S., Rush B., Raviola G., Saxena S., Thornicroft G., Yatham L.N. (2020). Mental health of communities during the COVID-19 pandemic. Can. J. Psychiatry.

[9] Panda P.K., Gupta J., Chowdhury S.R., Kumar R., Meena A.K., Madaan P., Sharawat I.K., Gulati S. (2021). Psychological and behavioral impact of lockdown and quarantine measures for COVID-19 pandemic on children, adolescents and caregivers: A systematic review and meta-analysis. J. Trop. Pediatr..

[10] Serafini G., Parmigiani B., Amerio A., Aguglia A., Sher L., Amore M. (2020). The psychological impact of COVID-19 on the mental health in the general population. QJM Mon. J. Associ Phys..

[11] Taquet M., Geddes J., Luciano S., Harrison P. (2022). Incidence and outcomes of eating disorders during the COVID-19 pandemic. Br. J. Psychiatry.

[12] Alimoradi Z., Broström A., Tsang H.W., Griffiths M.D., Haghayegh S., Ohayon M.M., Lin C.-Y., Pakpour A.H. (2021). Sleep problems during COVID-19 pandemic and its’ association to psychological distress: A systematic review and meta-analysis. EClinicalMedicine.

[13] Nobre P., Rosa P.J., Vasconcelos P., Tavares I., Carvalho J., Quinta-Gomes A., Moura C., Carrito M. (2022). Sexual health and the pandemic crisis: Testing the role of psychological vulnerability/protective factors on sexual functioning and sexual distress during a critical life period in Portugal. Arch. Sex. Behav..

[14] World Health Organization (2006). Defining Sexual Health: Report of a Technical Consultation on Sexual Health, 28–31 January 2002. https://www.who.int/health-topics/sexual-health#tab=tab_2.

[15] Ballester-Arnal R., Nebot-Garcia J.E., Ruiz-Palomino E., Giménez-García C., Gil-Llario M.D. (2021). “INSIDE” project on sexual health in Spain: Sexual life during the lockdown caused by COVID-19. Sex. Res. Soc. Policy.

[16] Pascoal P.M., Carvalho J., Raposo C.F., Almeida J., Beato A.F. (2021). The impact of COVID-19 on sexual health: A preliminary framework based on a qualitative study with clinical sexologists. Sex. Med..

[17] Lehmiller J.J., Garcia J.R., Gesselman A.N., Mark K.P. (2020). Less sex, but more sexual diversity: Changes in sexual behavior during the COVID-19 coronavirus pandemic. Leis. Sci..

[18] Skinta M.D., Sun A.H., Ryu D.M. (2020). The Impact of COVID-19 on the Lives of Sexual and Gender Minority People.

[19] Meyer I.H. (2003). Prejudice, social stress, and mental health in lesbian, gay, and bisexual populations: Conceptual issues and research evidence. Psychol. Bull..

[20] Goldey K.L., Cital M.N., Rodriguez S.C., Espinosa A., Barton E.A. (2022). Desire on lockdown? Sexual desire and COVID-19 stress among LGBTQ+ and cisgender, heterosexual college students. Psychol. Sex. Orientat. Gend. Divers..

[21] Salerno J.P., Devadas J., Pease M., Nketia B., Fish J.N. (2020). Sexual and gender minority stress amid the COVID-19 pandemic: Implications for LGBTQ young persons’ mental health and well-being. Public Health Rep..

[22] Kirana P.S., Gudeloglu A., Sansone A., Fode M., Reisman Y., Corona G., Burri A. (2020). E-sexual health: A position statement of the European Society for Sexual Medicine. Sex. Med..

[23] Davis F.D. (1989). Perceived usefulness, perceived ease of use, and user acceptance of information technology Manage. Inf. Syst. Q..

[24] Portela F., Santos M.F., Silva A., Rua F., Abelha A., Machado J. (2013). Adoption of Pervasive Intelligent Information Systems in Intensive Medicine. Procedia Technol..

[25] Mendes-Santos C., Weiderpass E., Santana R., Andersson G. (2020). Portuguese psychologists’ attitudes toward internet interventions: Exploratory cross-sectional study. JMIR Ment. Health.

[26] Hsieh H.F., Shannon S.E. (2005). Three approaches to qualitative content analysis. Qual. Health Res..

[27] Thomas D.R. (2006). A general inductive approach for analyzing qualitative evaluation data. Am. J. Eval..

[28] Lareau A. (2012). Using the Terms Hypothesis and Variable for Qualitative Work: A Critical Reflection. J. Marriage Fam..

[29] Cornwall A., Jewkes R. (1995). What is participatory research?. Soc. Sci. Med..

[30] World Medical Association (2013). Declaration of Helsinki: Ethical principles for medical research involving human subjects. JAMA.

[31] European Commission (2010). European Textbook on Ethics in Research.

[32] Vieira D.A., Meirinhos V. (2021). COVID-19 Lockdown in Portugal: Challenges, Strategies and Effects on Mental Health. Trends Psychol..

[33] Zhang Y., Wildemuth B.M., Wildemuth B. (2009). Qualitative Analysis of Content.

[34] Rapport F. (2010). Summative analysis: A qualitative method for social science and health research. Int. J. Qual. Methods.

[35] Cito G., Micelli E., Cocci A., Polloni G., Russo G.I., Coccia M.E., Simoncini T., Carini M., Minervini A., Natali A. (2021). The impact of the COVID-19 quarantine on sexual life in Italy. Urology.

[36] Masoudi M., Maasoumi R., Bragazzi N.L. (2022). Effects of the COVID-19 pandemic on sexual functioning and activity: A systematic review and meta-analysis. BMC Public Health.

[37] Boldrini T., Lomoriello A.S., Del Corno F., Lingiardi V., Salcuni S. (2020). Psychotherapy during COVID-19: How the clinical practice of Italian psychotherapists changed during the pandemic. Front. Psychol..

[38] Berger T. (2017). The therapeutic alliance in internet interventions: A narrative review and suggestions for future research. Psychother. Res..

[39] Soubelet-Fagoaga I., Arnoso-Martínez M., Guerendiain-Gabás I., Martínez-Moreno E., Ortiz G. (2021). (Tele)Work and Care during Lockdown: Labour and socio-familial restructuring in times of COVID-19. Int. J. Environ. Res. Public Health.

[40] Ramos J., Gómez A. (2020). ¿Por qué los retos de la conciliación en tiempos de COVID-19 son todavía mayores para las mujeres?. IvieExpress.

[41] Zhang Y., Wen C., Zhang Y., Luo X., Ma Z.F. (2021). The Impact of mental health and stress concerns on relationship and sexuality amidst the COVID-19 lockdown. J. Sex. Med..

[42] Szilagyi N., Olezeski C.L. (2020). Challenges in providing care for parents of transgender youth during the coronavirus pandemic. Smith Coll. Stud. Soc. Work.

[43] Durso L.E., Meyer I.H. (2013). Patterns and predictors of disclosure of sexual orientation to healthcare providers among lesbians, gay men, and bisexuals. Sex. Res. Soc. Policy.

[44] Carvalho J., Pascoal P.M. (2020). Challenges in the Practice of Sexual Medicine in the Time of COVID-19 in Portugal. J. Sex. Med..

